# A Metabolic-Related Gene Signature for Predicting Biochemical Recurrence After Radical Prostatectomy: An Integrative Analysis and Targeted Therapeutic Validation

**DOI:** 10.3390/ijms27114797

**Published:** 2026-05-26

**Authors:** Wankun Wang, Xiujuan Hong, Xiaoqi Wang, Ganpei Jiao, Hongjie Cai, Junxiang Zhao, Zhibing Wu, Jun Chen

**Affiliations:** 1 School of Medicine, Zhejiang University, Hangzhou 310058, China; wkwang@zju.edu.cn (W.W.);; 2 Second Clinical Medical College, Zhejiang Chinese Medical University, Hangzhou 310053, China

**Keywords:** prostate cancer, biochemical recurrence, metabolic reprogramming, prognostic model, machine learning, immune infiltration, molecular docking

## Abstract

Biochemical recurrence (BCR) after radical prostatectomy (RP) remains a major clinical challenge. Although metabolic reprogramming drives prostate cancer (PCa) progression, its predictive value for BCR and its interplay with the tumor immune microenvironment (TIME) remain incompletely understood. By integrating weighted gene co-expression network analysis (WGCNA) with machine learning, we identified four metabolic-related hub genes (*GDPD1*, *PLA2G7*, *PTGDS*, and *SRD5A2*) and developed an XGBoost-Cox model that accurately stratified BCR risk (training 5-year AUC: 0.858; validation 5-year AUC: 0.745). SHAP analysis enhanced the model’s interpretability, while immunohistochemistry (IHC) validated differential protein expression of these targets across 32 clinical specimens. Furthermore, immune profiling demonstrated that these genes are closely linked to M2 macrophage-mediated immunosuppression and altered T-cell infiltration. To translate these biomarkers into therapeutic targets, we employed in silico screening, molecular docking, and molecular dynamics simulations, identifying (-)-epigallocatechin gallate (EGCG) as a promising multi-target candidate. Subsequent in vitro assays confirmed that EGCG binds stably to GDPD1, PTGDS, and SRD5A2, effectively suppressing malignant PCa phenotypes and prostate-specific antigen (PSA) secretion. In summary, we established a robust and interpretable model for predicting BCR after RP, and our in vitro validation suggests that EGCG holds promise as a therapeutic agent to delay PCa progression.

## 1. Introduction

Prostate cancer (PCa) represents a formidable global health burden, ranking as the second most common malignancy and the fifth leading cause of cancer-specific death in the male population worldwide [1]. Although radical prostatectomy (RP) is the standard curative treatment for localized PCa, postoperative outcomes vary substantially among patients [2]. After RP, approximately 27–53% of patients develop biochemical recurrence (BCR), commonly defined as a prostate-specific antigen (PSA) level ≥ 0.2 ng/mL [3,4,5]. As the earliest indicator of treatment failure, BCR is associated with an increased risk of distant metastasis and mortality, yet its clinical course is highly heterogeneous [6]. While some patients rapidly progress to metastatic castration-resistant PCa (mCRPC), others follow a more indolent course [7]. Therefore, identifying the specific biomarkers that distinguish aggressive from indolent disease is critical for optimizing postoperative management.

Traditional clinicopathological parameters, such as the Gleason score and initial PSA level, cannot fully account for the complex biological networks that govern early recurrence. However, by integrating high-dimensional transcriptomic features, machine learning approaches have emerged as a powerful means of overcoming the limitations of these parameters in predicting PCa recurrence [8,9,10]. Specifically, transcriptomic subtyping provides a robust molecular basis for categorizing patients according to their risk of recurrence [11]. Building on these analytical frameworks, validated multi-gene signatures now provide a reliable means of assessing recurrence risk across diverse patient cohorts [12].

Metabolic reprogramming is a key driver of PCa progression [13]. PCa exhibits distinct metabolic dependencies, particularly on androgen signaling and lipid metabolism [14]. Dysregulated lipid and steroid biosynthesis are closely linked to tumor growth, aggressiveness, and resistance to therapy [15,16]. Beyond serving as prognostic biomarkers and therapeutic targets, specific metabolic biomarkers profoundly shape the tumor immune microenvironment (TIME) and drive immune cell polarization [17]. Although specific metabolites actively promote an immunosuppressive environment, the underlying regulators of these pathways and their functional roles in BCR remain poorly characterized [18].

This study integrated transcriptomics, weighted gene co-expression network analysis (WGCNA), and advanced statistical algorithms (elastic net and stepwise Cox) to identify a four-gene metabolic signature (*GDPD1*, *PLA2G7*, *PTGDS*, and *SRD5A2*) that predicts BCR of PCa. A BCR risk prediction model based on these hub genes was developed and validated. Immune infiltration profiling was conducted to elucidate the specific roles of these genes in reshaping the TIME. Additionally, molecular docking and molecular dynamics (MD) simulations confirmed the strong binding affinity of (-)-epigallocatechin gallate (EGCG) to these gene-encoded proteins, and in vitro experiments confirmed its targeted therapeutic potential. This study clarifies the potential link between metabolic reprogramming and the BCR of PCa, thereby providing new insights into the diagnosis and treatment of BCR.

## 2. Results

### 2.1. Identification of Differentially Expressed Genes (DEGs) and Functional Enrichment Analysis

**Figure 1:**
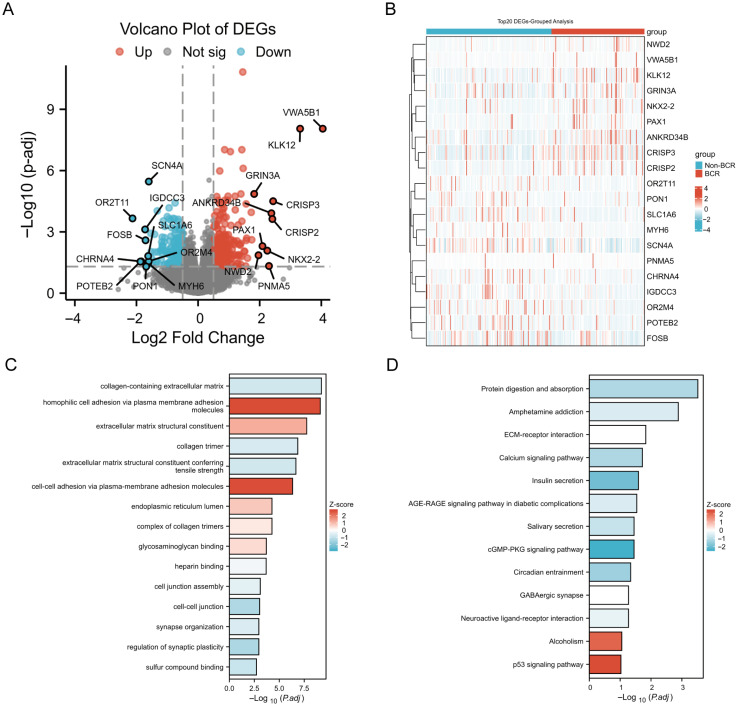

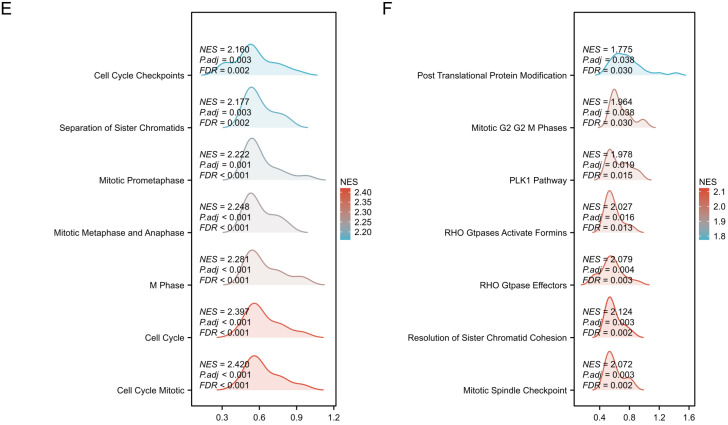
Identification and functional enrichment analysis of differentially expressed genes (DEGs) in patients with prostate cancer (PCa) with biochemical recurrence (BCR) versus non-BCR. (**A**) Volcano plot of DEGs comparing BCR and non-BCR groups (red: upregulated; blue: downregulated; the grey dashed line in the figure indicates that |Log2 fold change (FC)| > 0.5 and *p.adj* < 0.05). (**B**) Expression heatmap of the top 20 DEGs. (**C**,**D**) Bar charts summarizing the top enriched Gene Ontology (GO) terms (colored by Z-score) and Kyoto Encyclopedia of Genes and Genomes (KEGG) pathways. (**E**,**F**) Gene Set Enrichment Analysis (GSEA) ridge plots highlighting upregulated cell cycle, mitosis, and RHO GTPase pathways in the BCR group (FDR < 0.05).

To explore the transcriptomic changes associated with BCR in PCa, we systematically compared gene expression profiles between the BCR and non-BCR groups in the training cohort. Differential expression analysis identified a set of DEGs, including *VWA5B1*, *KLK12*, and *CRISP3*, which were significantly upregulated, and *OR2T11*, *FOSB*, and *PON1*, which were significantly downregulated (Figure 1A). Hierarchical clustering of the top 20 DEGs successfully separated BCR and non-BCR samples, reflecting significant changes in gene expression during disease recurrence (Figure 1B).

Functional enrichment analyses revealed that these DEGs are primarily associated with extracellular matrix (ECM) remodeling, cell adhesion, and the p53 signaling pathway (Figure 1C,D). Moreover, Gene Set Enrichment Analysis (GSEA) revealed that cell cycle-related networks were highly activated in the BCR group, including mitotic progression, spindle checkpoints, and PLK1/RHO GTPase signaling (Figure 1E,F). Collectively, these findings suggest that ECM remodeling and cell cycle dysregulation are critical to PCa progression and BCR.

### 2.2. Weighted Gene Co-Expression Network Analysis (WGCNA)

**Figure 2:**
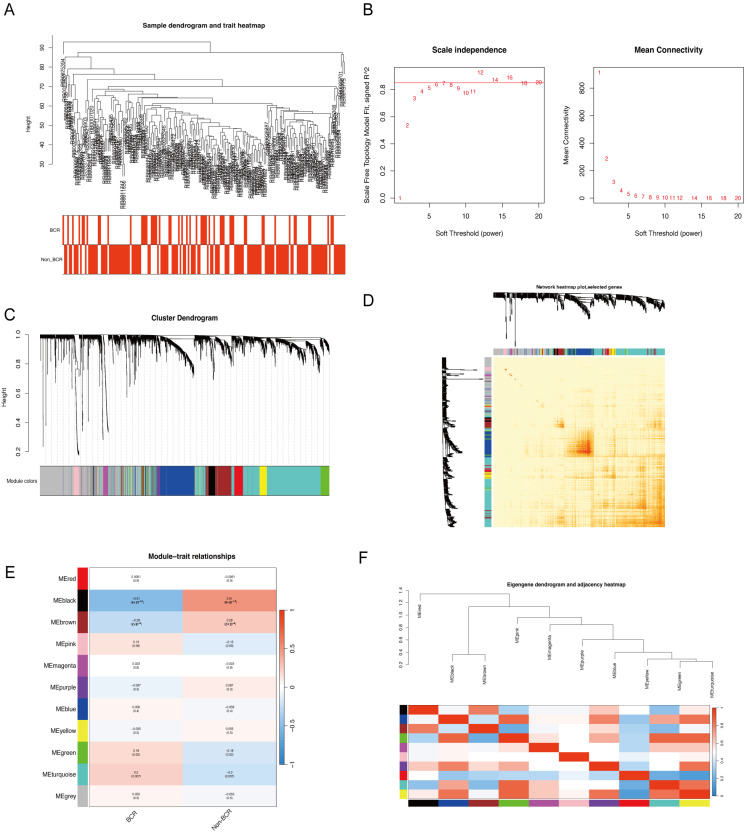

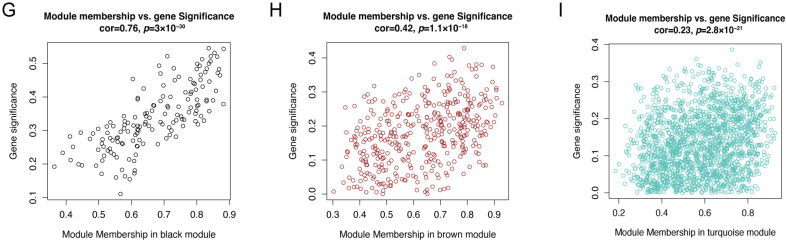
Weighted gene co-expression network analysis (WGCNA) identifies co-expression modules associated with BCR. (**A**) Sample clustering dendrogram of the training cohort. (**B**) Network topology analysis for soft-thresholding power selection in WGCNA. Numbers indicate different soft-thresholding power values; β = 12 was selected. (**C**,**D**) Gene hierarchical clustering dendrogram and corresponding module colors. (**E**) Heatmap of module–trait relationships. The black, brown, and turquoise modules were significantly correlated with BCR (|cor|≥ 0.2, *p* < 0.05; red and blue indicate positive and negative correlations). (**F**) The TOM heatmap illustrates the network’s structural integrity (red and blue indicate positive and negative correlations). (**G**–**I**) Scatterplots of GS versus MM reveal 2210 key module genes associated with BCR.

To investigate the underlying gene co-expression networks of BCR, WGCNA was used to identify key BCR-associated modules. First, a sample clustering analysis was performed on the training cohort, revealing no significant outliers (Figure 2A). Next, the scale-free topology fit index (R^2^ > 0.85) and mean connectivity were evaluated across a range of soft-thresholding powers, resulting in the selection of β = 12 to construct the co-expression network (Figure 2B). Using the dynamic tree-cut method, the genes were clustered and visually represented by distinct branch colors. This resulted in 11 significant co-expression modules (Figure 2C–E). Specifically, three modules—the black, brown, and turquoise modules—exhibited significant correlations with BCR (|cor| ≥ 0.2, *p* < 0.05) (Figure 2E). A topological overlap matrix (TOM) heatmap confirmed dense interconnectivity within modules and clear independence between them (Figure 2F). Ultimately, 2210 BCR-related genes were identified across these three modules. Scatterplots of gene significance (GS) versus module membership (MM) show their correlation with BCR (Figure 2G–I).

### 2.3. Identification and Functional Enrichment Analysis of Metabolic-Related Differentially Expressed Genes (MR-DEGs)

**Figure 3:**
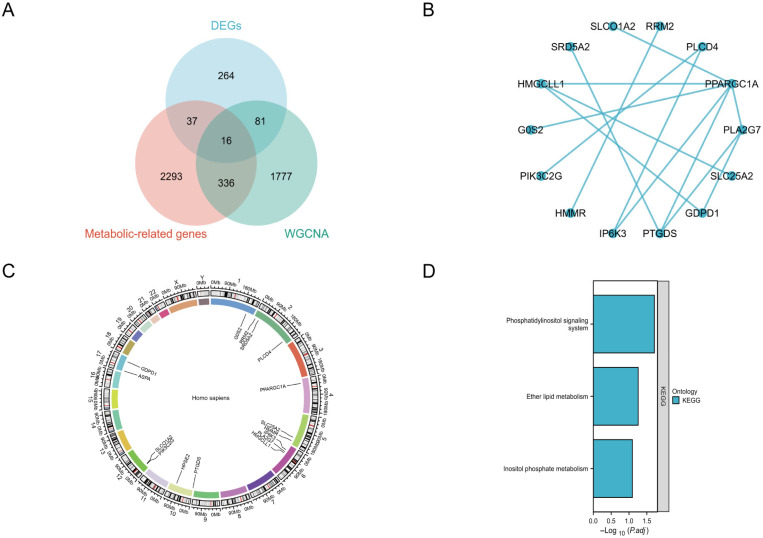

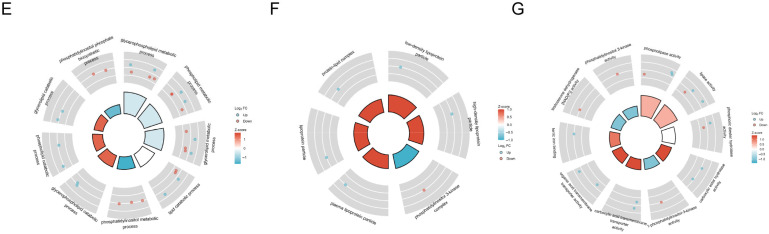
Identification and functional enrichment analysis of metabolic-related differentially expressed genes (MR-DEGs). (**A**) A Venn diagram showing the overlap of 16 MR-DEGs among the DEGs, the WGCNA BCR-associated module genes, and the MRGs. (**B**) A protein–protein interaction (PPI) network was constructed for the 16 MR-DEGs to highlight their internal interactions. (**C**) Chromosomal distribution of the 16 MR-DEGs, visualized in a circular format. (**D**) KEGG pathway enrichment analysis indicating the primary metabolic pathways associated with the MR-DEGs. (**E**–**G**) GO enrichment analysis of the MR-DEGs, detailing the enriched (**E**) biological processes (BP), (**F**) cellular components (CC), and (**G**) molecular functions (MF).

A total of 16 MR-DEGs were robustly identified by intersecting the DEGs, with the BCR-associated module genes identified via WGCNA and the metabolic-related genes (MRGs) (Figure 3A). A protein–protein interaction (PPI) network revealed that *PPARGC1A*, *PTGDS*, *PLA2G7*, and *HMGCLL1* were the primary interacting nodes (Figure 3B). Additionally, chromosomal mapping localized these genes predominantly to chromosomes 1, 2, 4, 5, 6, 10, 12, and 17 (Figure 3C).

### 2.4. Identification of Hub Genes and Validation of Protein Expression

**Figure 4:**
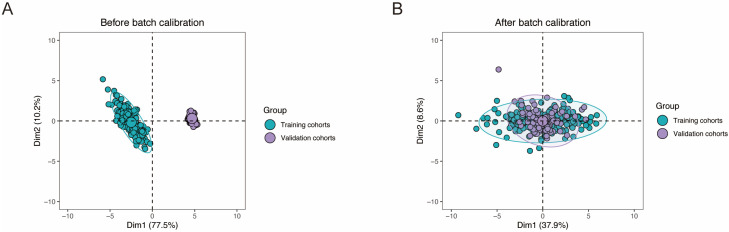

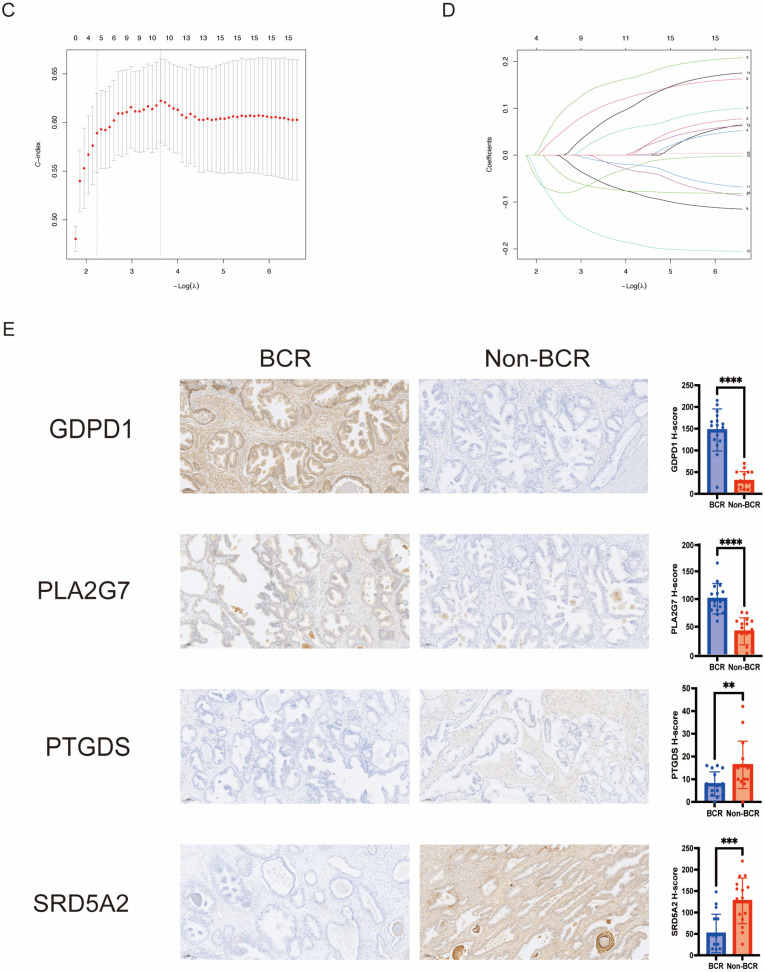
Identification and clinical validation of four metabolic-related hub genes in PCa. (**A**) Principal component analysis (PCA) plot of the groups before ComBat batch correction. (**B**) PCA plot of the groups after ComBat batch correction. (**C**,**D**) Elastic net logistic regression analysis for candidate gene selection illustrates the 10-fold cross-validation for optimal parameter selection and the corresponding coefficient profiles of the 16 MR-DEGs. (**E**) Representative IHC images and corresponding quantitative analysis of the four hub genes in tumor tissues from patients with BCR and non-BCR. (Data are presented as mean ± SD, ** *p* < 0.01, *** *p* < 0.001, **** *p* < 0.0001).

Before model construction, potential batch effects between the training and validation datasets were removed to ensure consistency across cohorts (Figure 4A,B). A two-step feature selection strategy was then applied to the 16 MR-DEGs to address multicollinearity. First, elastic net logistic regression identified six optimal predictive features associated with BCR status (Figure 4C,D). These results were combined with BCR-free survival data to perform a final stepwise Cox regression analysis, which identified four hub genes: *GDPD1*, *PLA2G7*, *PTGDS*, and *SRD5A2*.

To assess protein expression of these four hub genes in clinical specimens, immunohistochemistry (IHC) was performed on PCa tissues. Consistent with earlier transcriptomic profiles, IHC analysis revealed significant upregulation of GDPD1 and PLA2G7 and marked downregulation of PTGDS and SRD5A2 in samples from patients who experienced BCR compared with those without recurrence (Figure 4E). These results confirm the differential expression of these four hub genes in relation to BCR status and highlight their potential as biomarkers for predicting BCR in PCa patients.

### 2.5. Development and Interpretation of the Prognostic Model

**Figure 5:**
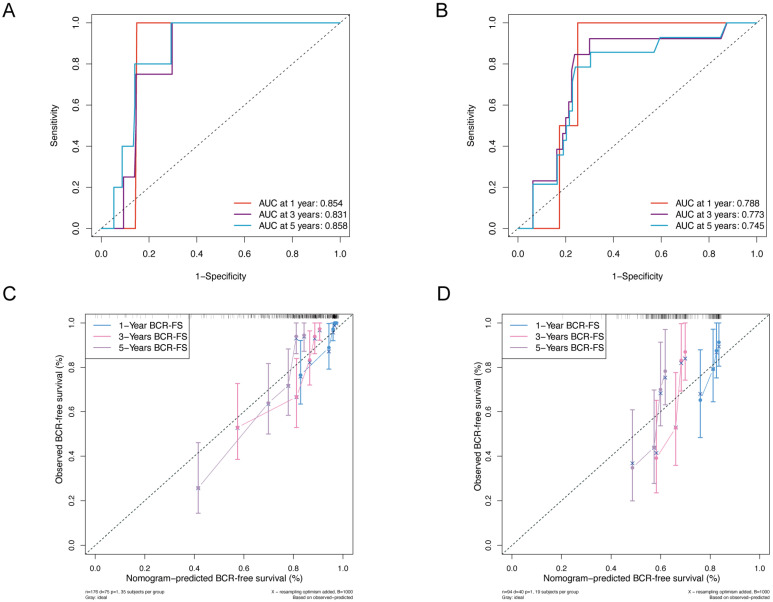

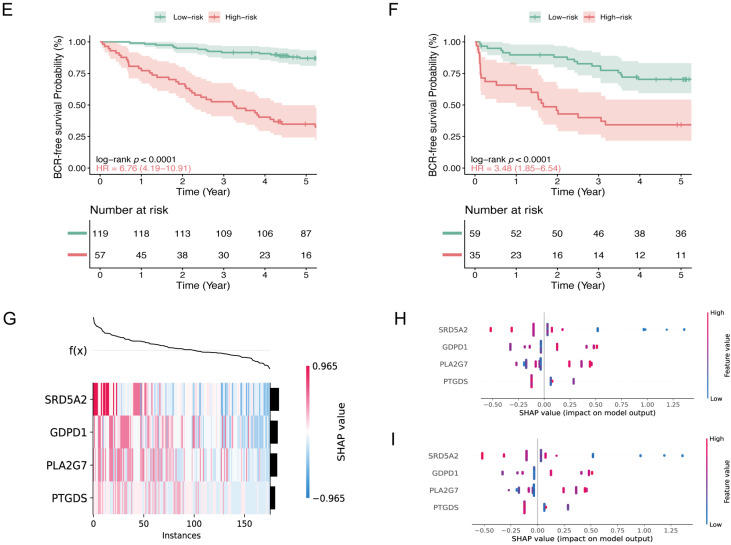
Performance evaluation and SHAP interpretation of the XGBoost model. (**A**,**B**) Time-dependent receiver operating characteristic (ROC) curves for the training (**A**) and validation (**B**) cohorts. (**C**,**D**) Calibration curves assessing predicted versus observed BCR probabilities in both cohorts. The marks above the curves represent the distribution of predicted probabilities. (**E**,**F**) Kaplan–Meier BCR-free survival analysis for patients stratified by risk score (log-rank *p* < 0.0001). (**G**–**I**) SHAP interpretability analysis, including a global heatmap (**G**) and beeswarm plots (**H**,**I**), delineating the impact of each hub gene’s transcript level on the risk score.

To explore nonlinear interactions among the transcript levels of these four hub genes, we constructed an XGBoost-Cox model to predict the BCR probability of PCa. Hyperparameters were optimized via a grid search with 10-fold cross-validation, and an independent prognostic risk score was calculated for each patient. The model demonstrated strong discrimination, yielding C-indices of 0.784 (95% CI: 0.738–0.830) and 0.680 (95% CI: 0.595–0.765) in the training and validation cohorts, respectively. Time-dependent receiver operating characteristic (ROC) analysis confirmed the model’s robust predictive accuracy, with 1-, 3-, and 5-year areas under the curve (AUCs) of 0.854, 0.831, and 0.858 in the training cohort, and 0.788, 0.773, and 0.745 in the validation cohort (Figure 5A,B). Additionally, calibration curves showed strong agreement between predicted and observed BCR events (Figure 5C,D).

Using an optimal riskScore cutoff of 0.7196 (determined by the minP method), patients were stratified into high- and low-risk groups. Kaplan–Meier analysis showed that high-risk patients had significantly worse BCR-free survival in both the training and validation cohorts (log-rank *p* < 0.0001 in both; Figure 5E,F). Finally, SHapley Additive exPlanations (SHAP) analysis effectively visualized the model’s biological interpretability, delineating each gene’s specific contribution to risk prediction (Figure 5G–I).

### 2.6. The Influence of Hub Genes on the Tumor Immune Microenvironment (TIME)

**Figure 6:**
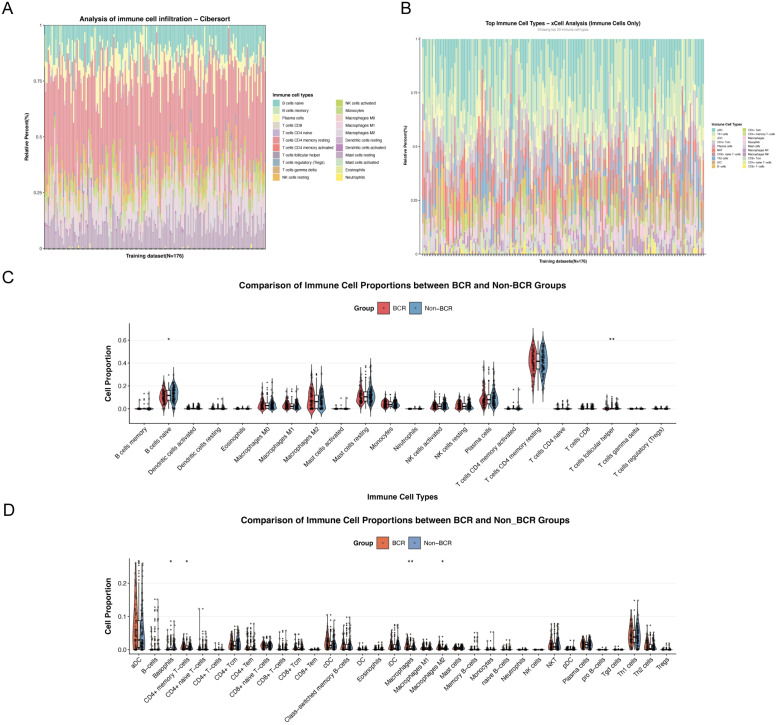

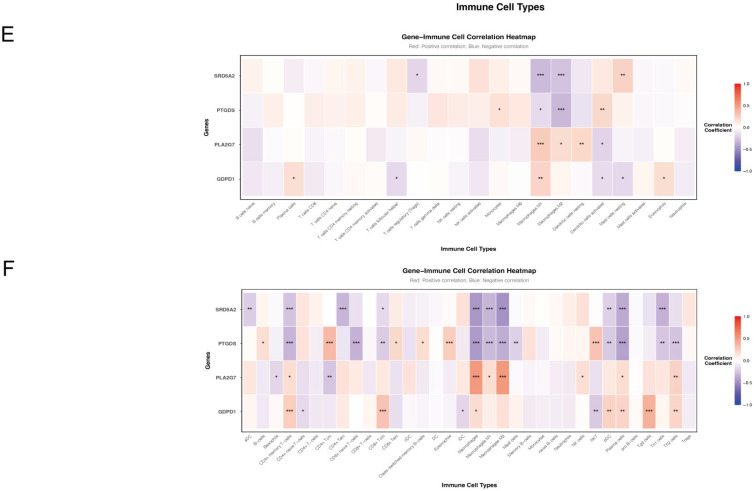
Correlation analysis between the hub genes and immune cell infiltration. (**A**,**B**) Overall distribution and relative proportions of immune cells in the training cohort, as evaluated using the CIBERSORT (**A**) and xCell (**B**) algorithms. (**C**,**D**) Comparison of specific immune cell infiltration abundances between the BCR and non-BCR groups, calculated using CIBERSORT (**C**) and xCell (**D**). (**E**,**F**) Correlation analyses illustrating the associations between the four hub genes and distinct immune cell types, based on CIBERSORT (**E**) and xCell (**F**) estimations (* *p* < 0.05, ** *p* < 0.01, *** *p* < 0.001).

The immune infiltration landscape of PCa was characterized using CIBERSORT and xCell (Figure 6A,B). Compared with the non-BCR group, patients who experienced BCR exhibited significant alterations in the TIME. Notably, the BCR group showed a substantial accumulation of general and M2 macrophages, as well as altered proportions of naive B cells, T follicular helper (Tfh) cells, activated dendritic cells (aDCs), basophils, and CD4+ memory T cells (Figure 6C,D). The enrichment of M2 macrophages suggests that the BCR group has an immunosuppressive microenvironment that promotes disease recurrence.

A correlation analysis was conducted between the expression levels of these four hub genes and the abundance of infiltrating immune cells to determine how they shape the TIME (Figure 6E,F). The detailed correlation coefficients and significance patterns were visualized using bubble-lollipop plots based on independent CIBERSORT (Figure S1) and xCell (Figure S2) analyses. Notably, these hub genes exhibited profound and complex connections with both innate and adaptive immune components. Beyond their significant associations with macrophage polarization, these hub genes were also significantly related to other critical immune subsets. Specifically, strong correlations were observed with distinct T cell populations, such as CD4+ memory T cells and central memory T cells (Tcm), as well as with innate effectors, such as DCs. These findings suggest that these hub genes drive BCR not only through a single cell type but also through systemic remodeling that coordinates complex innate and adaptive immune networks.

**Figure 7:**
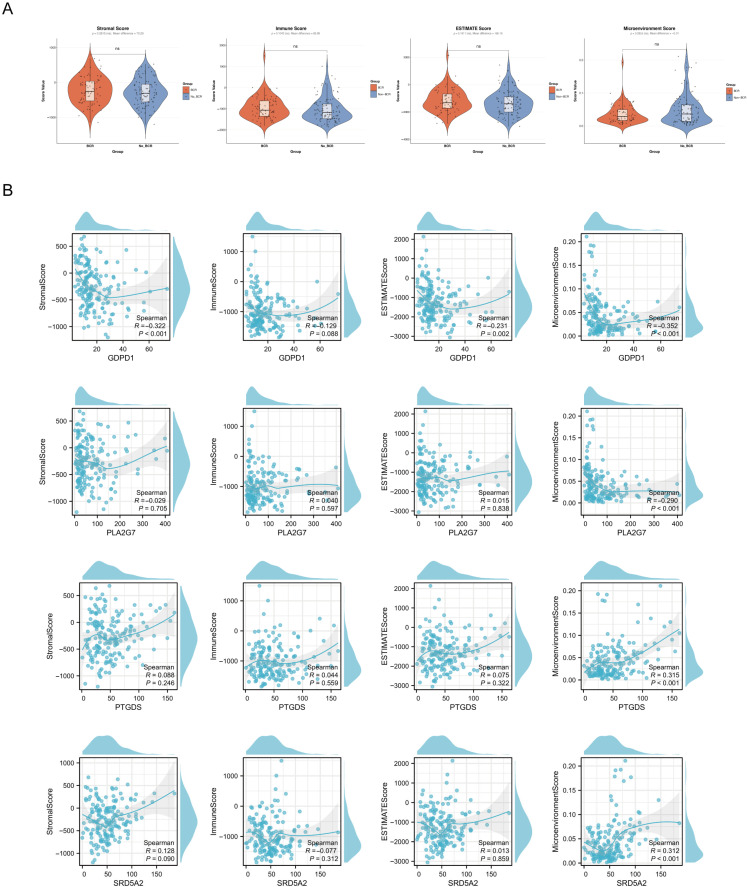
Comparison of TME scores between BCR and non-BCR groups and correlation analysis with the four hub genes. (**A**) Comparison of TME scores (Stromal, Immune, ESTIMATE, and Microenvironment) between the BCR and non-BCR groups; (**B**) Correlation analysis between the four hub genes and TME scores (ns, no significance).

ESTIMATE analysis revealed no significant differences in macroscopic tumor microenvironment (TME) scores (Stromal, Immune, ESTIMATE, and Microenvironment) between the BCR and non-BCR groups (Figure 7A). However, all four hub genes showed significant correlations with the MicroenvironmentScore. *GDPD1* also showed specific correlations with the StromalScore and ESTIMATEScore (Figure 7B).

### 2.7. In Silico Screening and Molecular Dynamics (MD) Simulations Validation of Targeted Therapeutics

Potential therapeutic compounds targeting the four hub genes were screened using the DSigDB database. The analysis identified two compounds capable of interacting with multiple targets: (-)-epigallocatechin gallate (EGCG, targeting *GDPD1*, *PTGDS*, and *SRD5A2*) and tetradioxin (targeting *PLA2G7*, *PTGDS*, and *SRD5A2*). However, tetradioxin was excluded from further validation due to its well-documented physiological toxicity. Thus, the natural catechin EGCG was chosen as the optimal candidate drug.

**Figure 8:**
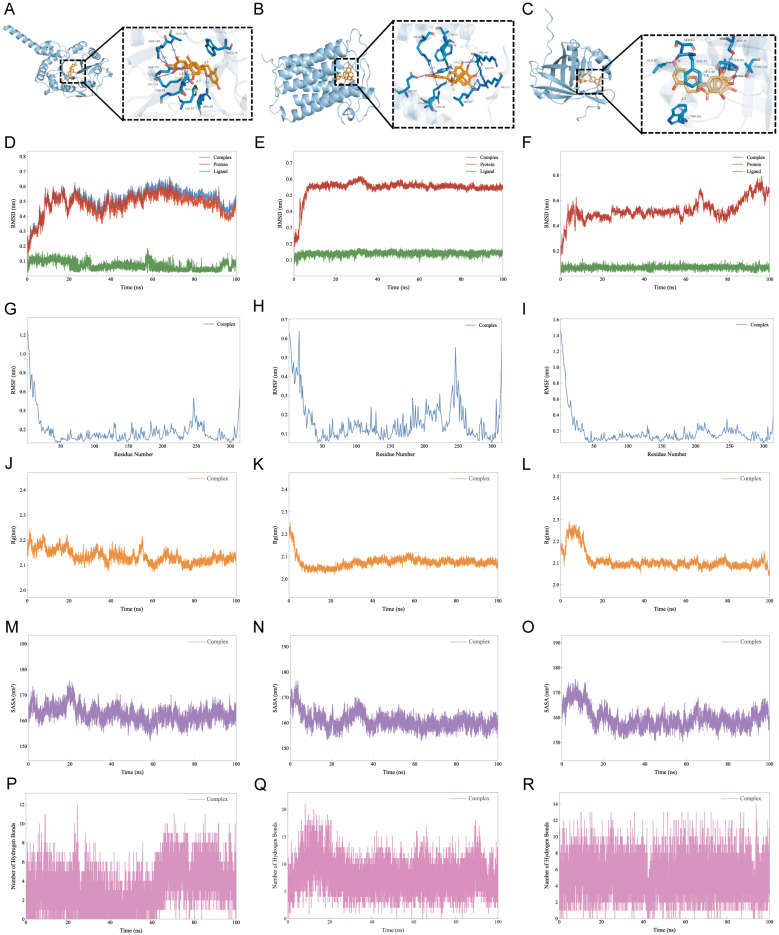

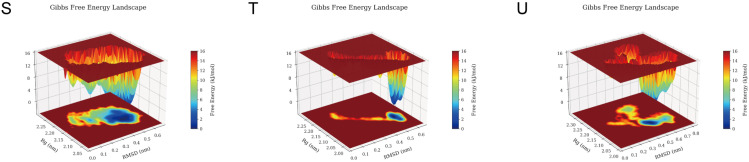
Molecular docking and molecular dynamics (MD) simulations of EGCG. The left, middle, and right columns present analyses of the EGCG-GDPD1, EGCG-PTGDS, and EGCG-SRD5A2 complexes, respectively. (**A**–**C**) 3D docking poses of EGCG within the catalytic active pocket of GDPD1 (**A**) and within the stabilizing non-active cavities of PTGDS (**B**) and SRD5A2 (**C**), the protein is shown in blue and EGCG is shown in yellow. (**D**–**R**) Structural dynamics over 100 ns MD simulations, including RMSD (**D**–**F**), RMSF (**G**–**I**), Rg (**J**–**L**), SASA (**M**–**O**), and the number of intermolecular hydrogen bonds (**P**–**R**). (**S**–**U**) Gibbs free energy landscapes (FEL) of the three complexes.

Molecular docking was subsequently performed to evaluate the spatial conformations and binding affinities of EGCG with the proteins encoded by these three hub genes. The docking results showed that EGCG successfully occupied the predicted active pocket of the upregulated risk target (GDPD1) and the non-active stabilizing cavities of the downregulated protective targets (PTGDS and SRD5A2). These interactions yielded low binding energies of −8.0, −8.2, and −12.0 kcal/mol, respectively (Figure 8A–C). These results suggest the presence of favorable intermolecular forces that establish optimal binding modes for both catalytic antagonism and structural stabilization.

Subsequent 100 ns MD simulations confirmed the conformational stability of these systems under physiological conditions. Structural metrics, including RMSD, RMSF, Rg, and SASA, showed that the systems equilibrated and became structurally compact throughout the trajectories (Figure 8D–O). The continuous presence of intermolecular hydrogen bonds (Figure 8P–R) and the convergence to global minima in Gibbs free energy landscapes (Figure 8S–U) also validated the stable binding of EGCG to its specific proteins. These findings suggest that EGCG may modulate the structure of these proteins, thereby counteracting the development and progression of PCa.

### 2.8. In Vitro Functional Validation of the Therapeutic Potential of EGCG

**Figure 9:**
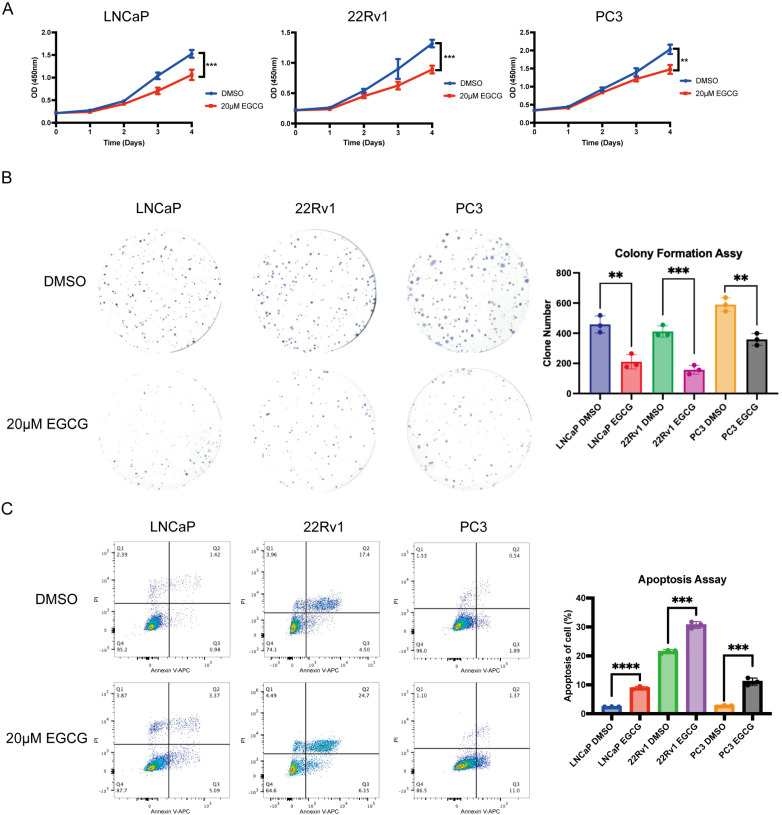
EGCG suppresses proliferation and induces apoptosis in PCa cells. (**A**) Cell proliferation curves for LNCaP, 22Rv1, and PC3 cells treated with DMSO or 20 μM EGCG over four days, assessed by CCK-8 assay. (**B**) Representative images and quantification of colony formation assays for the three cell lines. (**C**) Flow cytometry analysis of cellular apoptosis using Annexin V/PI staining (** *p* < 0.01, *** *p* < 0.001, **** *p* < 0.0001). Data are presented as mean ± SD from three independent experiments.

To functionally validate the therapeutic potential of EGCG, its in vitro efficacy was evaluated in three PCa cell lines (LNCaP, 22Rv1, and PC3). CCK-8 assays revealed a time-dependent suppression of cell viability following EGCG treatment across all lines (Figure 9A). Colony formation assays confirmed this anti-proliferative effect by showing reduced clonogenic capacity (Figure 9B). Furthermore, Annexin V/PI flow cytometry showed a significant induction of apoptosis in EGCG-treated cells (Figure 9C).

**Figure 10:**
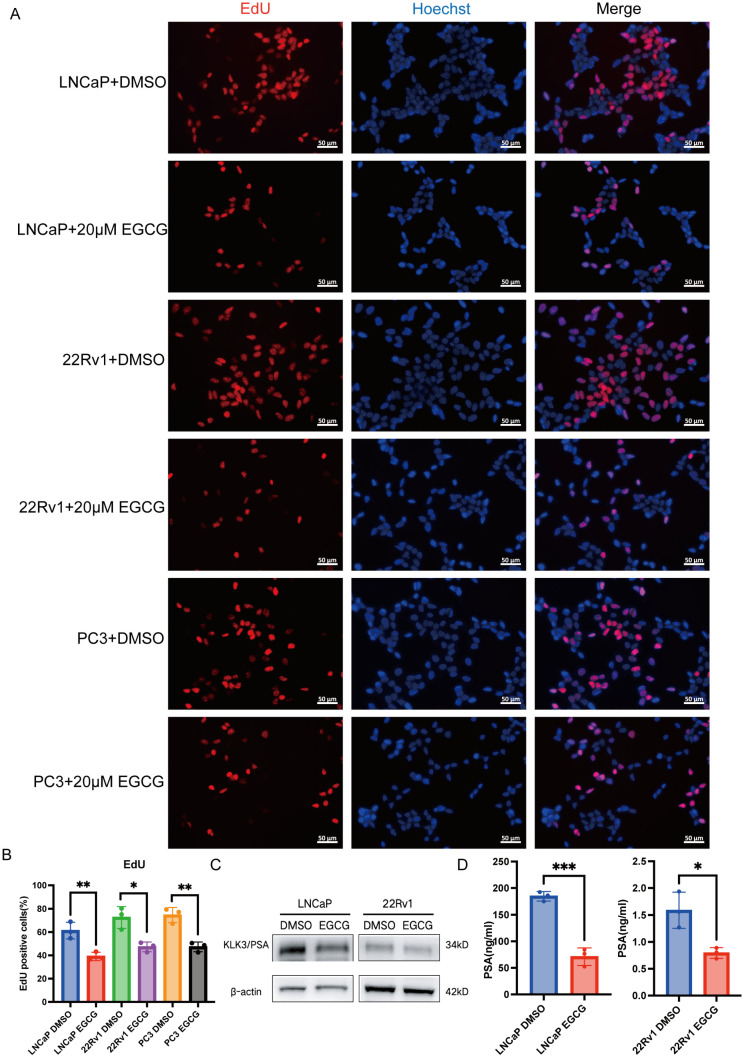
EGCG inhibits DNA synthesis and reduces PSA expression in PCa cells. (**A**,**B**) EdU incorporation assays evaluate active DNA synthesis in LNCaP, 22Rv1, and PC3 cells. Representative fluorescence images (**A**) and quantification of the proportion of EdU-positive cells (**B**). (**C**) Western blot analysis of intracellular PSA protein levels in LNCaP and 22Rv1 cells following EGCG treatment. (**D**) ELISA quantification of secreted PSA levels in the culture supernatant of LNCaP and 22Rv1 cells (* *p* < 0.05, ** *p* < 0.01, *** *p* < 0.001. Data are presented as the mean ± SD from three independent experiments).

The inhibition of cellular proliferation was further validated by EdU incorporation assays, which showed a reduced proportion of actively proliferating cells following EGCG exposure (Figure 10A,B). Since BCR is clinically characterized by rising prostate-specific antigen (PSA) levels, we evaluated the downstream regulatory effects of this targeted intervention in the AR-positive, PSA-secreting LNCaP and 22Rv1 cell lines. Western blot analysis confirmed downregulation of intracellular PSA protein expression (Figure 10C), and ELISA demonstrated a concurrent reduction in secreted PSA levels in the culture supernatant (Figure 10D). Taken together, these in vitro findings provide compelling functional evidence that EGCG suppresses PCa cell proliferation and survival and fundamentally represses PSA expression. This reinforces EGCG’s potential as an effective agent against BCR.

## 3. Discussion

BCR following RP represents a highly heterogeneous clinical phase in PCa, with patient trajectories ranging from indolent disease to lethal metastatic castration-resistant PCa (mCRPC) [19]. Consequently, precise postoperative risk stratification is imperative to optimize clinical management. However, conventional clinicopathological nomograms and static genomic profiling often fail to fully capture the dynamic metabolic and microenvironmental changes that drive early tumor recurrence [20,21]. To address this limitation, our research focuses on the unique metabolic reprogramming of PCa, exploring how specific metabolic changes mediate BCR and alter the TIME. By integrating transcriptomic analysis with machine learning, we identified a four-gene metabolic signature (*GDPD1*, *PLA2G7*, *PTGDS,* and *SRD5A2*) and developed an interpretable XGBoost-Cox model to accurately predict BCR. Crucially, we have clarified how these hub genes reshape the TIME, particularly by driving M2 macrophage polarization. Building on these mechanistic insights, we further explored whether these targets could be structurally modulated by specific compounds. Through in silico screening and in vitro validation, we demonstrated that the structural and functional states of these targets can be precisely modulated by the natural ligand EGCG. By directly binding to these targets, EGCG effectively suppresses the malignant phenotype. This indicates that these targets are not only valuable for predicting BCR risk in patients but also serve as practical therapeutic targets.

Metabolic reprogramming is widely recognized as a hallmark of PCa progression. Specifically, dysregulation of lipid and phospholipid metabolism distinguishes PCa from most other malignancies, which rely predominantly on aerobic glycolysis [15,16]. The four hub genes in our prognostic model strongly reflect this distinct metabolic dependency.

*GDPD1* (Glycerophosphodiester phosphodiesterase domain containing 1) is a critical hub gene identified in our signature. It encodes an essential enzyme in choline phospholipid metabolism. It belongs to the glycerophosphodiester phosphodiesterase family and catalyzes the hydrolysis of deacylated glycerophospholipids into glycerol phosphate and alcohol. This enzymatic activity is crucial for maintaining optimal membrane fluidity and supporting lipid signaling [22]. In highly proliferative malignancies, enzymes in the GDPD family, such as GDPD1, are often dysregulated to accelerate membrane biosynthesis. This process is essential for sustaining aggressive tumor growth [23]. Our study revealed that GDPD1 is a metabolic enzyme that promotes BCR occurrence, as it was significantly upregulated in the BCR cohort. Additionally, our immune infiltration analysis revealed that elevated GDPD1 expression was significantly associated with increased infiltration of immune cells, including CD4+ memory T cells, CD8+ Tcm cells, and γδ T cells. These results suggest that excessive lipid turnover driven by GDPD1 promotes tumor proliferation and significantly alters the composition of the TME. EGCG acts as a competitive inhibitor by binding directly to the enzyme’s active site, thereby inhibiting GDPD1 activity. Specifically, our docking models showed that EGCG occupies the catalytic core pocket through direct interactions with residues Glu-72, Asp-74, and His-87 [24]. These residues are essential for the subsequent hydrolase activity characteristic of GDPD1. By physically occluding this site, EGCG directly reduces substrate accessibility and effectively inhibits GDPD1’s enzymatic function.

*PLA2G7* (phospholipase A2 group VII) encodes a key metabolic enzyme that hydrolyzes oxidized phospholipids, generating highly active proinflammatory lipid mediators, such as lysophosphatidylcholine (LPC) [25]. Previous research has shown that this phospholipase is associated with higher Gleason scores, increased cell migration, and shorter BCR-free survival [26]. Our research also confirmed that PLA2G7 is significantly overexpressed in BCR tissues. Furthermore, our immune infiltration analysis revealed that higher PLA2G7 expression is closely associated with the presence of specific immune cell populations, particularly the immunosuppressive M2 macrophage phenotype. These findings suggest that the lipid byproducts produced by PLA2G7 promote M2 macrophage polarization. This creates an immunosuppressive microenvironment that accelerates post-operative recurrence. Although our in silico screening revealed that EGCG effectively targets GDPD1, it did not predict a direct interaction with PLA2G7. This suggests that although PLA2G7 remains a crucial prognostic biomarker, an alternative inhibitor is needed due to its distinct structural pocket. Therefore, effective targeting of the complex metabolic landscape of PCa may ultimately require combinatorial therapeutic strategies.

However, our research found that *PTGDS* expression was significantly downregulated in patients with BCR. PTGDS, the protein encoded by this gene, is a key enzyme that catalyzes the production of prostaglandin D2. It also functions as a critical tumor suppressor in PCa [27]. Its downregulation has been shown to induce endothelial-to-mesenchymal transition (EndMT), thereby increasing vascular permeability and creating a microenvironment that promotes tumor recurrence and metastasis [28]. Clinically, the absence of PTGDS is often associated with excessive activation of AR signaling, which further exacerbates the malignant phenotype of PCa [29]. Notably, our analysis of immune infiltration revealed a strong negative correlation between PTGDS expression and the abundance of macrophages (M1 and M2 phenotypes), plasma cells, and Th2 cells. Mechanistically, chronic inflammation mediated by Th2 cells and tumor-associated macrophages actively drives PCa progression [30]. This negative correlation suggests that low PTGDS expression in BCR tissues may promote chronic inflammation. This inflammation leads to the abnormal accumulation of these tumorigenic inflammatory cells, creating conditions that promote tumor recurrence. Because PTGDS functions as a tumor suppressor, we investigated whether EGCG binding might compromise its activity. Our molecular docking analysis revealed that, rather than occupying the catalytic Cys-65 core, EGCG binds to a distinct peripheral pocket containing Tyr-33, Trp-53, and Arg-94 [31,32]. This suggests that EGCG does not inhibit the enzyme but instead stabilizes its functional conformation. Furthermore, our molecular dynamics (MD) simulation revealed that PTGDS became more stable upon binding with EGCG. These results suggest that EGCG’s targeting of PTGDS can protect the enzyme from degradation and help maintain immune balance in the microenvironment.

The *SRD5A2* gene encodes the enzyme that converts testosterone into the more potent dihydrotestosterone (DHT). DHT is essential for maintaining normal prostate homeostasis [33]. In our prognostic model, SRD5A2 expression was significantly lower in patients with BCR. Clinically, loss of SRD5A2 independently predicts BCR after RP [34]. Consistent with the protective role of SRD5A2, our immune infiltration analysis revealed a strong negative correlation between its expression and the accumulation of macrophages (M1 and M2 phenotypes) and other inflammatory cells. These findings suggest that the metabolic disorder caused by downregulation of SRD5A2 exacerbates the development of a pro-tumor inflammatory microenvironment. Given EGCG’s role in maintaining prostatic homeostasis, we investigated whether its binding might impair SRD5A2 function. Interestingly, our molecular docking analysis showed that EGCG does not inhibit this essential enzyme. Although SRD5A2’s catalytic activity strictly depends on active site residues such as Glu-57 and Arg-114, our docking model indicates that EGCG binds to a distinct allosteric cavity containing Ser-45, Leu-48, and Trp-54. This binding completely avoids the main active site [35]. Furthermore, our MD simulation revealed that SRD5A2 is structurally similar to PTGDS and that it becomes more stable upon binding to EGCG. This suggests that EGCG may protect SRD5A2 from degradation.

Our findings suggest that BCR is associated with systemic remodeling of the TIME. The accumulation of M2 macrophages in the BCR group aligns with previous studies identifying these cells as independent predictors of poor postoperative prognosis [36,37]. This transition to an immunosuppressive state may be driven by potential metabolic changes within the tumor. Specifically, altered lipid metabolism has been shown to polarize macrophages toward a pro-tumorigenic phenotype. This state helps the tumor evade immune detection and promotes recurrence [38,39]. In addition to innate immunity, the metabolic remodeling we observed is associated with changes in the infiltration of CD4+ memory T cells and T follicular helper (Tfh) cells. This indicates that this metabolic remodeling also affects adaptive immunity [40]. These alterations in innate and adaptive immunity suggest that metabolic reprogramming affects the TIME as a whole rather than a single cell type. Targeting these metabolic biomarkers with EGCG could reverse this immunosuppressive environment and provide practical strategies to prevent or delay the onset of BCR [41].

We developed a prognostic model using the XGBoost-Cox proportional hazards framework to translate these metabolic biomarkers into a practical tool for risk stratification. Traditional statistical models, such as standard Cox regression, often oversimplify the high-dimensional nature of transcriptomic data, limiting their ability to model the nonlinear biological networks that drive tumor recurrence [42,43]. XGBoost effectively overcomes these limitations by using a gradient boosting framework. It inherently models complex nonlinear interactions and is uniquely suited to handling right-censored clinical survival data, thereby improving prognostic accuracy [44]. The established model achieved favorable AUC and C-index values in both cohorts, indicating improved predictive performance compared with traditional approaches. Importantly, SHAP analysis clarified the XGBoost model’s decision-making process, revealing the biological significance of each gene in predicting patient prognosis [45]. This visualization shows how each gene affects a patient’s risk score, providing the interpretability necessary for translation into personalized clinical management.

EGCG, the primary bioactive polyphenol in green tea, has been extensively studied for its pleiotropic anti-tumor properties [46]. It antagonizes AR signaling by inhibiting histone acetyltransferase activity, thereby preventing AR translocation to the nucleus [47]. Epigenetically, EGCG suppresses DNA methyltransferase activity, thereby reactivating tumor suppressors such as GSTP1 [48]. Furthermore, it counteracts PCa metabolic reprogramming via the AMPK and PI3K/Akt/mTOR pathways [49,50,51]. Importantly, EGCG disrupts lipid synthesis by targeting fatty acid synthase [52], and suppresses tumor-associated macrophages within the TIME [53]. Our MD results elucidate this microenvironmental regulation. They reveal that EGCG directly inhibits GDPD1 and structurally stabilizes the protective enzymes PTGDS and SRD5A2. This targeted, concurrent modulation likely restricts lipid signals that drive macrophage infiltration, thereby demonstrating its multi-target potential [41,54].

Despite these promising preclinical findings, a realistic strategy is needed to translate EGCG into clinical practice. A well-documented limitation of EGCG is its low systemic bioavailability and rapid in vivo degradation [55]. Furthermore, EGCG may pose safety risks, including potential hepatotoxicity and unpredictable off-target effects, at high pharmacological doses [56,57]. Advanced delivery platforms, such as lipid–polymer hybrid nanoparticles (LPHNPs), offer a promising avenue to overcome these pharmacokinetic barriers and enhance tumor-specific targeting [58]. Clinically, EGCG is unlikely to serve as a standalone treatment for established BCR. Instead, its most feasible application is as a preventive agent in the adjuvant setting following RP [59,60]. Additionally, EGCG shows significant promise as part of a combination therapy regimen, potentially synergizing with novel AR pathway inhibitors, such as enzalutamide, to comprehensively delay PCa progression and reduce therapeutic resistance [61].

Although our four-gene metabolic signature has clinical value, we must address the challenges posed by tumor heterogeneity and sampling bias. Because PCa is usually multifocal, local tissue sampling may not accurately reflect the entire genomic landscape [62]. To mitigate this issue, we used WGCNA to identify stable hub genes. Network-based module analyses have been proven to be fundamentally more robust to transcriptomic noise and spatial heterogeneity than randomly selected individual biomarkers [63,64]. Future validations require a multi-modal approach to overcome heterogeneity and sampling bias. Integrating spatial transcriptomics with single-cell RNA sequencing (scRNA-seq) will clarify the in situ metabolic architecture and the clonal diversity of individual cells [65]. Additionally, longitudinal liquid biopsies can capture the systemic transcriptomic landscape, thereby effectively bypassing the spatial limitations of conventional biopsies [66].

Our study established a novel, interpretable, machine learning-derived metabolic-related gene signature to predict BCR in PCa. This signature highlights critical prognostic models, immune microenvironment remodeling, and targeted therapeutic interventions. Nevertheless, several limitations should be acknowledged. First, this retrospective model requires prospective validation [67]. Second, the specific pathways that drive immune regulation require further functional exploration via co-culture systems [68,69]. Finally, the in vivo efficacy and safety of EGCG must be rigorously tested in patient-derived xenograft (PDX) models before clinical translation [70].

## 4. Materials and Methods

### 4.1. Data Acquisition and Preprocessing

Gene expression profiles and corresponding clinical information for PCa patients were obtained from the Gene Expression Omnibus (GEO) database. The GSE220095 dataset (platform GPL16791) was used as the training cohort and included 176 patients (75 with BCR and 101 without BCR within five years). The GSE70769 dataset (platform GPL10558) was used for external validation and included 94 patients (40 with BCR and 54 without BCR within five years). The clinical characteristics of PCa patients from the GEO database are listed in Table S1. Additionally, formalin-fixed paraffin-embedded (FFPE) tissue sections from 32 patients (16 with BCR and 16 without BCR within five years) who underwent RP were retrospectively collected between 2020 and 2025 at the Department of Urology, Affiliated Zhejiang Hospital, Zhejiang University School of Medicine. This study was approved by the Institutional Ethics Committee (Approval No. ZJHIRB-2026-001K).

**Figure 11:**
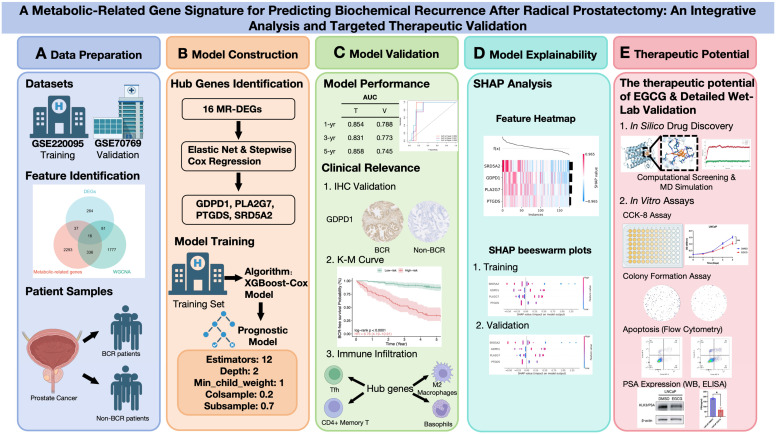
Flowchart of the study design (* *p* < 0.05, *** *p* < 0.001).

After acquiring the data, DEGs between the BCR and non-BCR groups in the training cohort were identified using the “limma” package (version 3.60.6) in R (version 4.4.1) [71]. |Log_2_ fold change (FC)| > 0.5 and p.adj < 0.05 were used as the thresholds for identifying DEGs. To visualize differences in gene expression, volcano plots and hierarchical clustering heatmaps of the top 20 DEGs were generated using the “ggplot2” (version 4.0.1) and “pheatmap” (version 1.0.13) packages. An overview of the study procedure is provided in Figure 11.

### 4.2. Pathway Enrichment Analysis

To annotate the biological functions and signaling pathways of the identified DEGs, GO and KEGG pathway analyses, as well as GSEA, were performed using the ‘clusterProfiler’ package (version 4.14.6) in R.

### 4.3. WGCNA

The R package “WGCNA” (version 1.73) was used to construct a co-expression network using the top 25% of variance-ranked genes [72]. The optimal soft-thresholding power (β) was determined using the pickSoftThreshold function based on the scale-free network topology criterion. Modules containing at least 50 genes were identified using the one-step network construction approach, with unassigned genes grouped into the gray module. Pearson correlation between module eigengenes and clinical traits was used to identify modules associated with BCR. Furthermore, the relationships between individual genes and BCR were assessed using gene significance (GS) and module membership (MM).

### 4.4. Identification and Screening of Metabolism-Related Hub Genes

A total of 2682 MRGs were extracted from the Hallmark gene sets in the Molecular Signatures Database (MSigDB) [73]. Overlapping these MRGs with the previously identified DEGs and the BCR-associated WGCNA module genes yielded 16 MR-DEGs, which were visualized via a Venn diagram.

To identify metabolic-related hub genes, the 16 MR-DEGs underwent a two-step selection process. The “sva” package in R was used to implement the ComBat algorithm, which removed batch effects between the training and validation cohorts, enabling downstream modeling. First, elastic net logistic regression with cross-validation was used to address multicollinearity and reduce the features to six candidates. Next, a multivariable stepwise Cox regression analysis identified four hub genes (*GDPD1*, *PLA2G7*, *PTGDS,* and *SRD5A2*) associated with BCR-free survival. These four hub genes were ultimately included in the final prognostic model.

### 4.5. Construction of PPI Network

The STRING database (https://string-db.org/, accessed on 13 December 2025) was used to create the PPI network for the MR-DEGs. A minimum interaction score of 0.4 (medium confidence) was set to ensure data reliability. Subsequently, the network interaction data were exported and visualized using Cytoscape (version 3.10.3).

### 4.6. Establishment, Validation, and Interpretation of the Prognostic Model

A Cox regression model based on XGBoost was constructed using the transcriptomic profiles of the four hub genes to predict BCR. The model’s hyperparameters were optimized using a grid search combined with ten-fold cross-validation. Based on the optimized model, a risk score (riskScore) was calculated for each patient. The predictive performance was evaluated using time-dependent ROC and calibration curves. Patients in the training cohort were stratified into high- and low-risk groups using an optimal cutoff identified by the minimum *p*-value (minP) method. Kaplan–Meier analysis was performed to compare BCR-free survival between patients in the high- and low-risk groups. We subsequently verified the model’s predictive accuracy and risk stratification in an independent validation cohort.

Furthermore, SHAP analysis was used to interpret the XGBoost-Cox model [44]. SHAP values were calculated to quantify each hub gene’s contribution to the individual riskScore. The distribution, directional impact, and overall importance of these genes across both cohorts were visualized with SHAP feature heatmaps and beeswarm plots.

### 4.7. Analysis of Immune Infiltration

The relative proportions of immune cell populations in the PCa microenvironment were estimated using the CIBERSORT and xCell algorithms. Additionally, immune, stromal, microenvironment, and ESTIMATE scores were computed for the BCR and non-BCR groups using the “estimate” R package. Finally, Spearman correlation analysis was used to assess associations between the four hub genes and both the differentially enriched immune cells and the microenvironment scores.

### 4.8. Identification of Candidate Drugs

Potential therapeutic drugs targeting the four hub genes were identified using the Drug Signatures Database (DSigDB) [74]. Compounds with high-confidence associations to the hub genes were ranked by enrichment strength and retained as potential therapeutic candidates for subsequent molecular modeling.

### 4.9. Molecular Docking and MD Simulations

Ligand 3D structures from PubChem and target proteins were prepared by removing crystallographic water and optimizing protonation states. Molecular docking was performed with AutoDock Tools v1.5.7 at the predicted active site. The conformation with the lowest binding energy was selected for subsequent MD simulations. MD simulations of the optimal docking complex were performed in GROMACS 2025 using the AMBER99SB force field. The system was solvated in a TIP3P cubic water box (12 Å clearance) and neutralized with 0.154 M NaCl. Following steepest descent energy minimization, the system underwent 100 ps of NVT (300 K) and NPT (1 bar) equilibration. Finally, a 100 ns production run (2 fs time step) was executed, saving trajectories every 10 ps.

### 4.10. Cell Culture

The human PCa cell lines LNCaP, 22Rv1, and PC3 were obtained from the American Type Culture Collection (ATCC, Manassas, USA). Cells were cultured in RPMI-1640 medium (Thermo Fisher Scientific, Waltham, USA; C11875) supplemented with 10% fetal bovine serum (FBS) and 1% penicillin-streptomycin at 37 °C in a humidified atmosphere containing 5% CO_2_.

### 4.11. Cell Viability Assay

Cell viability was assessed using the CCK-8 assay (Biosharp, Hefei, China; BL1055C) according to the manufacturer’s protocol. Briefly, cells were seeded into 96-well plates at 3000–5000 cells per well and allowed to adhere overnight. After treatment, 10 µL of CCK-8 reagent was added to each well. After an additional 2 h of incubation at 37 °C, absorbance was measured at 450 nm with a microplate reader.

### 4.12. Colony Formation Assay

Cells were seeded into six-well plates at 1000 cells per well. After treatment, cells were cultured for 10–14 days, with the culture medium replaced every 3–4 days. The resulting colonies were fixed with 4% paraformaldehyde, stained with 0.5% crystal violet, and counted. Clusters with ≥50 cells were defined as positive colonies.

### 4.13. Apoptosis Assay

Apoptosis was evaluated using the Annexin V/PI Apoptosis Detection Kit (Vazyme, Nanjing, China; A214-02). After treatment, cells were stained with Annexin V and propidium iodide (PI) for 15 min in the dark, then analyzed by flow cytometry.

### 4.14. EdU Incorporation Assay

Cell proliferation was evaluated using the Click-iT EdU-594 Assay Kit (Biosharp, Hefei, China; BL917A). After a 48 h treatment with DMSO or 20 μM EGCG, cells were incubated with 10 μM EdU at 37 °C for 2 h. Cells were then fixed with 4% paraformaldehyde, permeabilized with 0.1% Triton X-100 (Biosharp, Hefei, China; 1139ML100), and stained with the Click-iT cocktail (Click-iT EdU-594 Assay Kit, Biosharp, Hefei, China; BL917A) and Hoechst. Finally, EdU-positive cells were imaged by fluorescence microscopy, and the percentage of proliferating cells was quantified by calculating the ratio of EdU-stained cells to the total number of Hoechst-labeled nuclei using ImageJ (version 1.54, National Institutes of Health, Bethesda, MD, USA).

### 4.15. Western Blot

Total protein was extracted using RIPA buffer supplemented with protease inhibitors. Equal amounts of protein were separated by SDS-PAGE and transferred to PVDF membranes. The membranes were blocked with 5% nonfat milk at room temperature for 1 h, followed by an overnight incubation at 4 °C with primary antibodies against KLK3/PSA (Proteintech, Wuhan, China; 10679-1-AP) and β-actin (Servicebio, Beijing, China; GB12001-100), which served as a loading control for normalization. After washing, membranes were incubated with HRP-conjugated secondary antibodies at room temperature for 1 h, and protein bands were visualized using enhanced chemiluminescence.

### 4.16. Enzyme-Linked Immunosorbent Assay

PSA levels in cell culture supernatants were quantified using a commercial human PSA ELISA kit (Elabscience, Wuhan, China; E-EL-H0091) according to the manufacturer’s protocol. Absorbance was measured at 450 nm using a microplate reader, and absolute PSA concentrations were calculated from the standard curve.

### 4.17. IHC

Paraffin-embedded sections (4–5 μm) were deparaffinized and rehydrated. Then, they underwent microwave-assisted antigen retrieval in a citrate buffer (pH 6.0). Endogenous peroxidase was quenched with 3% hydrogen peroxide, and nonspecific binding was blocked with 5% normal serum. Sections were incubated overnight at 4 °C with the following primary antibodies: SRD5A2 (Affinity, Changzhou, China; #DF8416), GDPD1 (Proteintech, Wuhan, China; 27861-1-AP), PTGDS (Proteintech, Wuhan, China; 10754-2-AP), and PLA2G7 (Proteintech, Wuhan, China; 15526-1-AP). After PBS washes, slides were incubated with an HRP-conjugated secondary antibody, developed with DAB, and counterstained with hematoxylin. Sections were dehydrated, mounted, and scanned using an automated slide scanner (KFBIO, Ningbo, China; KF-PRO-120).

### 4.18. Statistical Analysis

Data are presented as mean ± SD. Analyses were performed using R (v 4.4.1) and GraphPad Prism 10. Two-group and multi-group comparisons were assessed using Student’s *t*-test (or a Mann–Whitney U test) and one-way ANOVA, respectively. BCR-free survival was analyzed using the Kaplan–Meier method and the log-rank test. A two-tailed *p* < 0.05 was considered significant (* *p* < 0.05, ** *p* < 0.01, *** *p* < 0.001 and **** *p* < 0.0001).

## 5. Conclusions

In this study, we developed a prognostic signature based on four metabolic-related hub genes (*GDPD1*, *PLA2G7*, *PTGDS*, and *SRD5A2*) that accurately predicts BCR and characterizes the immune microenvironment in PCa. Furthermore, EGCG was confirmed to selectively bind these targets and was validated in vitro to effectively suppress malignant phenotypes. These findings improve our understanding of metabolic reprogramming in PCa and provide a solid theoretical foundation for translating these targets into feasible therapeutic strategies to prevent BCR.

## Data Availability

The data presented in this study are available in GEO at https://www.ncbi.nlm.nih.gov/geo/, accessed on 1 December 2025, reference numbers GSE220095 and GSE70769. These data were derived from the following resources available in the public domain: GEO datasets GSE220095 (https://www.ncbi.nlm.nih.gov/geo/query/acc.cgi?acc=GSE220095, accessed on 1 December 2025) and GSE70769 (https://www.ncbi.nlm.nih.gov/geo/query/acc.cgi?acc=GSE70769, accessed on 1 December 2025).

## Supplementary Materials

The following supporting information can be downloaded at: https://www.mdpi.com/article/10.3390/ijms27114797/s1.

## Abbreviations

The following abbreviations are used in this manuscript:

BCR	Biochemical recurrence
RP	Radical prostatectomy
PCa	Prostate cancer
TIME	Tumor immune microenvironment
WGCNA	Weight gene co-expression network analysis
IHC	Immunohistochemistry
EGCG	(-)-Epigallocatechin gallate
PSA	Prostate-specific antigen
mCRPC	Metastatic castration-resistant prostate cancer
MD	Molecular dynamics
DEGs	Differentially expressed genes
ECM	Extracellular matrix
GSEA	Gene set enrichment analysis
GO	Gene ontology
BP	Biological processes
CC	Cellular components
MF	Molecular functions
KEGG	Kyoto encyclopedia of genes and genomes
TOM	Topological overlap matrix
GS	Gene significance
MM	Module membership
MRGs	Metabolic-related genes
MR-DEGs	Metabolic-related differentially expressed genes
PPI	Protein–protein interaction
PCA	Principal component analysis
CI	C-index
ROC	Receiver operating characteristic
AUC	Area under the curve
SHAP	SHapley Additive exPlanations
Tfh	T follicular helper
aDC	Activated dendritic cells
Tcm	Central memory T cells
TME	Tumor microenvironment
AR	Androgen receptor
ELISA	Enzyme-Linked Immunosorbent Assay
EndMT	Endothelial-to-mesenchymal transition
DHT	Dihydrotestosterone
PDX	Patient-derived xenograft
FFPE	Formalin-fixed paraffin-embedded
FC	Fold change
MSigDB	Molecular Signatures Database
DSigDB	Drug Signatures Database
FBS	Fetal bovine serum
PI	Propidium iodide

## References

[1] Bray F., Laversanne M., Sung H., Ferlay J., Siegel R.L., Soerjomataram I., Jemal A. (2024). Global cancer statistics 2022: GLOBOCAN estimates of incidence and mortality worldwide for 36 cancers in 185 countries. CA Cancer J. Clin..

[2] Mottet N., van den Bergh R.C.N., Briers E., Van den Broeck T., Cumberbatch M.G., De Santis M., Fanti S., Fossati N., Gandaglia G., Gillessen S. (2021). EAU-EANM-ESTRO-ESUR-SIOG Guidelines on Prostate Cancer-2020 Update. Part 1: Screening, Diagnosis, and Local Treatment with Curative Intent. Eur. Urol..

[3] Van den Broeck T., van den Bergh R.C.N., Arfi N., Gross T., Moris L., Briers E., Cumberbatch M., De Santis M., Tilki D., Fanti S. (2019). Prognostic Value of Biochemical Recurrence Following Treatment with Curative Intent for Prostate Cancer: A Systematic Review. Eur. Urol..

[4] Cornford P., van den Bergh R.C.N., Briers E., Van den Broeck T., Cumberbatch M.G., De Santis M., Fanti S., Fossati N., Gandaglia G., Gillessen S. (2021). EAU-EANM-ESTRO-ESUR-SIOG Guidelines on Prostate Cancer. Part II-2020 Update: Treatment of Relapsing and Metastatic Prostate Cancer. Eur. Urol..

[5] Artibani W., Porcaro A.B., De Marco V., Cerruto M.A., Siracusano S. (2018). Management of Biochemical Recurrence after Primary Curative Treatment for Prostate Cancer: A Review. Urol. Int..

[6] Shore N.D., Moul J.W., Pienta K.J., Czernin J., King M.T., Freedland S.J. (2024). Biochemical recurrence in patients with prostate cancer after primary definitive therapy: Treatment based on risk stratification. Prostate Cancer Prostatic Dis..

[7] Antonarakis E.S., Feng Z., Trock B.J., Humphreys E.B., Carducci M.A., Partin A.W., Walsh P.C., Eisenberger M.A. (2012). The natural history of metastatic progression in men with prostate-specific antigen recurrence after radical prostatectomy: Long-term follow-up. BJU Int..

[8] Wang Y., Zhu H., Ren J., Ren M. (2025). Integrative machine learning models predict prostate cancer diagnosis and biochemical recurrence risk: Advancing precision oncology. NPJ Digit. Med..

[9] Sato K., Sakamoto S., Saito S., Shibata H., Yamada Y., Takeuchi N., Goto Y., Tomokazu S., Imamura Y., Ichikawa T. (2024). Time-dependent personalized prognostic analysis by machine learning in biochemical recurrence after radical prostatectomy: A retrospective cohort study. BMC Cancer.

[10] Zhang T., Ide H., Lu J., Lu Y., China T., Nagata M., Hachiya T., Horie S. (2025). Predicting Biochemical Recurrence After Robot-Assisted Prostatectomy with Interpretable Machine Learning Model. J. Clin. Med..

[11] Zhang X., Johannessen B., Kidd S.G., Bogaard M., Stranger A., Axcrona U., Axcrona K., Skotheim R.I. (2026). Cell type composition in bulk prostate cancer tissue is a prognostic biomarker. Neoplasia.

[12] Lin B.B., Huang Q., Yan B., Liu M., Zhang Z., Lei H., Huang R., Dong J.T., Pang J. (2024). An 18-gene signature of recurrence-associated endothelial cells predicts tumor progression and castration resistance in prostate cancer. Br. J. Cancer.

[13] Pavlova N.N., Thompson C.B. (2016). The Emerging Hallmarks of Cancer Metabolism. Cell Metab..

[14] Mah C.Y., Nassar Z.D., Swinnen J.V., Butler L.M. (2020). Lipogenic effects of androgen signaling in normal and malignant prostate. Asian J. Urol..

[15] Sena L.A., Denmeade S.R. (2021). Fatty Acid Synthesis in Prostate Cancer: Vulnerability or Epiphenomenon?. Cancer Res..

[16] Bader D.A., McGuire S.E. (2020). Tumour metabolism and its unique properties in prostate adenocarcinoma. Nat. Rev. Urol..

[17] De Martino M., Rathmell J.C., Galluzzi L., Vanpouille-Box C. (2024). Cancer cell metabolism and antitumour immunity. Nat. Rev. Immunol..

[18] Watson M.J., Delgoffe G.M. (2022). Fighting in a wasteland: Deleterious metabolites and antitumor immunity. J. Clin. Investig..

[19] Preisser F., Chun F.K.H., Pompe R.S., Heinze A., Salomon G., Graefen M., Huland H., Tilki D. (2019). Persistent Prostate-Specific Antigen After Radical Prostatectomy and Its Impact on Oncologic Outcomes. Eur. Urol..

[20] Lamy P.J., Allory Y., Gauchez A.S., Asselain B., Beuzeboc P., de Cremoux P., Fontugne J., Georges A., Hennequin C., Lehmann-Che J. (2018). Prognostic Biomarkers Used for Localised Prostate Cancer Management: A Systematic Review. Eur. Urol. Focus.

[21] Abida W., Cyrta J., Heller G., Prandi D., Armenia J., Coleman I., Cieslik M., Benelli M., Robinson D., Van Allen E.M. (2019). Genomic correlates of clinical outcome in advanced prostate cancer. Proc. Natl. Acad. Sci. USA.

[22] Xie Y., Ella K.M., Gibbs T.C., Yohannan M.E., Knoepp S.M., Balijepalli P., Meier G.P., Meier K.E. (2024). Characterization of Lysophospholipase D Activity in Mammalian Cell Membranes. Cells.

[23] Glunde K., Bhujwalla Z.M., Ronen S.M. (2011). Choline metabolism in malignant transformation. Nat. Rev. Cancer.

[24] Chang P.A., Shao H.B., Long D.X., Sun Q., Wu Y.J. (2008). Isolation, characterization and molecular 3D model of human GDE4, a novel membrane protein containing glycerophosphodiester phosphodiesterase domain. Mol. Membr. Biol..

[25] Yang X., Tu Y., Liang N., Li L., Zhang J., Xu J., Li C. (2025). Lp-PLA2 in the cancer landscape: From molecular mechanisms to therapeutic potential (Review). Int. J. Oncol..

[26] Vainio P., Lehtinen L., Mirtti T., Hilvo M., Seppanen-Laakso T., Virtanen J., Sankila A., Nordling S., Lundin J., Rannikko A. (2011). Phospholipase PLA2G7, associated with aggressive prostate cancer, promotes prostate cancer cell migration and invasion and is inhibited by statins. Oncotarget.

[27] Chen B., Guo L., Wang L., Wu P., Zheng X., Tan C., Xie N., Sun X., Zhou M., Huang H. (2024). Leveraging cell death patterns to predict metastasis in prostate adenocarcinoma and targeting PTGDS for tumor suppression. Sci. Rep..

[28] Omori K., Morikawa T., Kunita A., Nakamura T., Aritake K., Urade Y., Fukayama M., Murata T. (2018). Lipocalin-type prostaglandin D synthase-derived PGD(2) attenuates malignant properties of tumor endothelial cells. J. Pathol..

[29] Thompson V.C., Day T.K., Bianco-Miotto T., Selth L.A., Han G., Thomas M., Buchanan G., Scher H.I., Nelson C.C., Australian Prostate Cancer B. (2012). A gene signature identified using a mouse model of androgen receptor-dependent prostate cancer predicts biochemical relapse in human disease. Int. J. Cancer.

[30] Boibessot C., Molina O., Lachance G., Tav C., Champagne A., Neveu B., Pelletier J.F., Pouliot F., Fradet V., Bilodeau S. (2022). Subversion of infiltrating prostate macrophages to a mixed immunosuppressive tumor-associated macrophage phenotype. Clin. Transl. Med..

[31] Kumasaka T., Aritake K., Ago H., Irikura D., Tsurumura T., Yamamoto M., Miyano M., Urade Y., Hayaishi O. (2009). Structural basis of the catalytic mechanism operating in open-closed conformers of lipocalin type prostaglandin D synthase. J. Biol. Chem..

[32] Irikura D., Kumasaka T., Yamamoto M., Ago H., Miyano M., Kubata K.B., Sakai H., Hayaishi O., Urade Y. (2003). Cloning, expression, crystallization, and preliminary X-ray analysis of recombinant mouse lipocalin-type prostaglandin D synthase, a somnogen-producing enzyme. J. Biochem..

[33] Wang Z., Gu B., Sharkey C., Ge R., Olumi A.F. (2026). SRD5A2 and emerging therapies in androgen-driven disorders. Nat. Rev. Urol..

[34] Audet-Walsh E., Bellemare J., Nadeau G., Lacombe L., Fradet Y., Fradet V., Huang S.P., Bao B.Y., Douville P., Girard H. (2011). SRD5A polymorphisms and biochemical failure after radical prostatectomy. Eur. Urol..

[35] Xiao Q., Wang L., Supekar S., Shen T., Liu H., Ye F., Huang J., Fan H., Wei Z., Zhang C. (2020). Structure of human steroid 5alpha-reductase 2 with the anti-androgen drug finasteride. Nat. Commun..

[36] Zhang Q., Xia J., Wang Y., Zhang J., Ji C., Cong R., Wang Y., Song N. (2019). Tumor infiltrating M2 macrophages could predict biochemical recurrence of localized prostate cancer after radical prostatectomy. Exp. Cell Res..

[37] Liu R., Lu J., Liu J., Liao Y., Guo Y., Shi P., Wang Z., Wang H., Lai J. (2025). Macrophages in prostate cancer: Dual roles in tumor progression and immune evasion. J. Transl. Med..

[38] Siltari A., Syvala H., Lou Y.R., Gao Y., Murtola T.J. (2022). Role of Lipids and Lipid Metabolism in Prostate Cancer Progression and the Tumor’s Immune Environment. Cancers.

[39] Zhang F., Liu W., Meng F., Jiang Q., Tang W., Liu Z., Lin X., Xue R., Zhang S., Dong L. (2024). Inhibiting PLA2G7 reverses the immunosuppressive function of intratumoral macrophages and augments immunotherapy response in hepatocellular carcinoma. J. Immunother. Cancer.

[40] Tan J., Jin X., Zhao R., Wei X., Liu Y., Kong X. (2015). Beneficial effect of T follicular helper cells on antibody class switching of B cells in prostate cancer. Oncol. Rep..

[41] McLarty J., Bigelow R.L., Smith M., Elmajian D., Ankem M., Cardelli J.A. (2009). Tea polyphenols decrease serum levels of prostate-specific antigen, hepatocyte growth factor, and vascular endothelial growth factor in prostate cancer patients and inhibit production of hepatocyte growth factor and vascular endothelial growth factor in vitro. Cancer Prev. Res..

[42] Rajkomar A., Dean J., Kohane I. (2019). Machine Learning in Medicine. N. Engl. J. Med..

[43] Topol E.J. (2019). High-performance medicine: The convergence of human and artificial intelligence. Nat. Med..

[44] Lundberg S.M., Erion G., Chen H., DeGrave A., Prutkin J.M., Nair B., Katz R., Himmelfarb J., Bansal N., Lee S.I. (2020). From Local Explanations to Global Understanding with Explainable AI for Trees. Nat. Mach. Intell..

[45] Lipkova J., Chen R.J., Chen B., Lu M.Y., Barbieri M., Shao D., Vaidya A.J., Chen C., Zhuang L., Williamson D.F.K. (2022). Artificial intelligence for multimodal data integration in oncology. Cancer Cell..

[46] Singh B.N., Shankar S., Srivastava R.K. (2011). Green tea catechin, epigallocatechin-3-gallate (EGCG): Mechanisms, perspectives and clinical applications. Biochem. Pharmacol..

[47] Lee Y.H., Kwak J., Choi H.K., Choi K.C., Kim S., Lee J., Jun W., Park H.J., Yoon H.G. (2012). EGCG suppresses prostate cancer cell growth modulating acetylation of androgen receptor by anti-histone acetyltransferase activity. Int. J. Mol. Med..

[48] Pandey M., Shukla S., Gupta S. (2010). Promoter demethylation and chromatin remodeling by green tea polyphenols leads to re-expression of GSTP1 in human prostate cancer cells. Int. J. Cancer.

[49] Li H.L., Huang Y., Zhang C.N., Liu G., Wei Y.S., Wang A.B., Liu Y.Q., Hui R.T., Wei C., Williams G.M. (2006). Epigallocathechin-3 gallate inhibits cardiac hypertrophy through blocking reactive oxidative species-dependent and -independent signal pathways. Free Radic. Biol. Med..

[50] Zhou Y., Huang S., Guo Y., Ran M., Shan W., Chen W.H., Tam K.Y. (2023). Epigallocatechin gallate circumvents drug-induced resistance in non-small-cell lung cancer by modulating glucose metabolism and AMPK/AKT/MAPK axis. Phytother. Res..

[51] Hwang J.T., Ha J., Park I.J., Lee S.K., Baik H.W., Kim Y.M., Park O.J. (2007). Apoptotic effect of EGCG in HT-29 colon cancer cells via AMPK signal pathway. Cancer Lett..

[52] Brusselmans K., De Schrijver E., Heyns W., Verhoeven G., Swinnen J.V. (2003). Epigallocatechin-3-gallate is a potent natural inhibitor of fatty acid synthase in intact cells and selectively induces apoptosis in prostate cancer cells. Int. J. Cancer.

[53] Li D., Cao D., Sun Y., Cui Y., Zhang Y., Jiang J., Cao X. (2024). The roles of epigallocatechin gallate in the tumor microenvironment, metabolic reprogramming, and immunotherapy. Front. Immunol..

[54] Bettuzzi S., Brausi M., Rizzi F., Castagnetti G., Peracchia G., Corti A. (2006). Chemoprevention of human prostate cancer by oral administration of green tea catechins in volunteers with high-grade prostate intraepithelial neoplasia: A preliminary report from a one-year proof-of-principle study. Cancer Res..

[55] Mereles D., Hunstein W. (2011). Epigallocatechin-3-gallate (EGCG) for clinical trials: More pitfalls than promises?. Int. J. Mol. Sci..

[56] Mazzanti G., Menniti-Ippolito F., Moro P.A., Cassetti F., Raschetti R., Santuccio C., Mastrangelo S. (2009). Hepatotoxicity from green tea: A review of the literature and two unpublished cases. Eur. J. Clin. Pharmacol..

[57] Younes M., Aggett P., Aguilar F., Crebelli R., Dusemund B., Filipic M., Frutos M.J., Galtier P., Gott D., EFSA Panel on Food Additives and Nutrient Sources added to Food (ANS) (2018). Scientific opinion on the safety of green tea catechins. EFSA J..

[58] Gajbhiye K.R., Salve R., Narwade M., Sheikh A., Kesharwani P., Gajbhiye V. (2023). Lipid polymer hybrid nanoparticles: A custom-tailored next-generation approach for cancer therapeutics. Mol. Cancer.

[59] Kallifatidis G., Hoy J.J., Lokeshwar B.L. (2016). Bioactive natural products for chemoprevention and treatment of castration-resistant prostate cancer. Semin. Cancer Biol..

[60] Lecumberri E., Dupertuis Y.M., Miralbell R., Pichard C. (2013). Green tea polyphenol epigallocatechin-3-gallate (EGCG) as adjuvant in cancer therapy. Clin. Nutr..

[61] Morrissey C., Brown M., O’Sullivan J., Weathered N., Watson R.W., Tenniswood M. (2007). Epigallocatechin-3-gallate and bicalutamide cause growth arrest and apoptosis in NRP-152 and NRP-154 prostate epithelial cells. Int. J. Urol..

[62] Boutros P.C., Fraser M., Harding N.J., de Borja R., Trudel D., Lalonde E., Meng A., Hennings-Yeomans P.H., McPherson A., Sabelnykova V.Y. (2015). Spatial genomic heterogeneity within localized, multifocal prostate cancer. Nat. Genet..

[63] van Dam S., Vosa U., van der Graaf A., Franke L., de Magalhaes J.P. (2018). Gene co-expression analysis for functional classification and gene-disease predictions. Brief. Bioinform..

[64] Barabasi A.L., Oltvai Z.N. (2004). Network biology: Understanding the cell’s functional organization. Nat. Rev. Genet..

[65] Song H., Weinstein H.N.W., Allegakoen P., Wadsworth M.H., Xie J., Yang H., Castro E.A., Lu K.L., Stohr B.A., Feng F.Y. (2022). Single-cell analysis of human primary prostate cancer reveals the heterogeneity of tumor-associated epithelial cell states. Nat. Commun..

[66] Casanova-Salas I., Athie A., Boutros P.C., Del Re M., Miyamoto D.T., Pienta K.J., Posadas E.M., Sowalsky A.G., Stenzl A., Wyatt A.W. (2021). Quantitative and Qualitative Analysis of Blood-based Liquid Biopsies to Inform Clinical Decision-making in Prostate Cancer. Eur. Urol..

[67] Simon R.M., Paik S., Hayes D.F. (2009). Use of archived specimens in evaluation of prognostic and predictive biomarkers. J. Natl. Cancer Inst..

[68] Chen S., Zhu G., Yang Y., Wang F., Xiao Y.T., Zhang N., Bian X., Zhu Y., Yu Y., Liu F. (2021). Single-cell analysis reveals transcriptomic remodellings in distinct cell types that contribute to human prostate cancer progression. Nat. Cell Biol..

[69] Comito G., Giannoni E., Segura C.P., Barcellos-de-Souza P., Raspollini M.R., Baroni G., Lanciotti M., Serni S., Chiarugi P. (2014). Cancer-associated fibroblasts and M2-polarized macrophages synergize during prostate carcinoma progression. Oncogene.

[70] Lin D., Wyatt A.W., Xue H., Wang Y., Dong X., Haegert A., Wu R., Brahmbhatt S., Mo F., Jong L. (2014). High fidelity patient-derived xenografts for accelerating prostate cancer discovery and drug development. Cancer Res..

[71] Ritchie M.E., Phipson B., Wu D., Hu Y., Law C.W., Shi W., Smyth G.K. (2015). limma powers differential expression analyses for RNA-sequencing and microarray studies. Nucleic Acids Res..

[72] Langfelder P., Horvath S. (2008). WGCNA: An R package for weighted correlation network analysis. BMC Bioinform..

[73] Subramanian A., Tamayo P., Mootha V.K., Mukherjee S., Ebert B.L., Gillette M.A., Paulovich A., Pomeroy S.L., Golub T.R., Lander E.S. (2005). Gene set enrichment analysis: A knowledge-based approach for interpreting genome-wide expression profiles. Proc. Natl. Acad. Sci. USA.

[74] Yoo M., Shin J., Kim J., Ryall K.A., Lee K., Lee S., Jeon M., Kang J., Tan A.C. (2015). DSigDB: Drug signatures database for gene set analysis. Bioinformatics.

