# Supplementary material for: A Metabolic-Related Gene Signature for Predicting Biochemical Recurrence After Radical Prostatectomy: An Integrative Analysis and Targeted Therapeutic Validation
Source: Int J Mol Sci. 2026 May 26;27(11):4797. doi: 10.3390/ijms27114797 (PMC13257006; doi:10.3390/ijms27114797)
Supplement: Supplementary file 1 [file ijms-27-04797-s001.zip › ijms-4336091-supplementary.pdf]

Supplementary Material

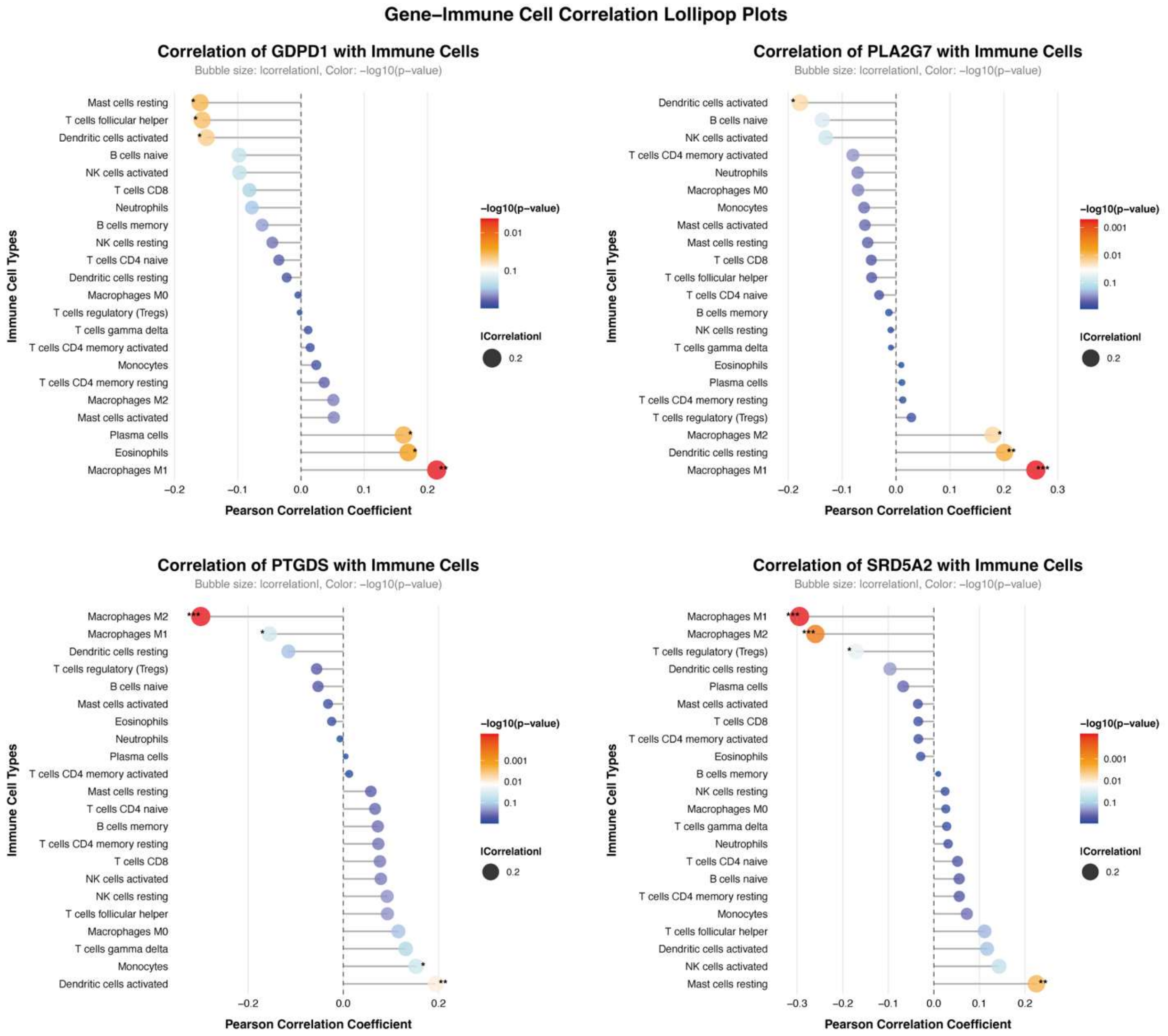

Figure S1. Correlation analysis of four metabolism-related hub genes with immune cells using the CIBERSORT method. ($*p < 0.05$, $**p < 0.01$, $***p < 0.001$).

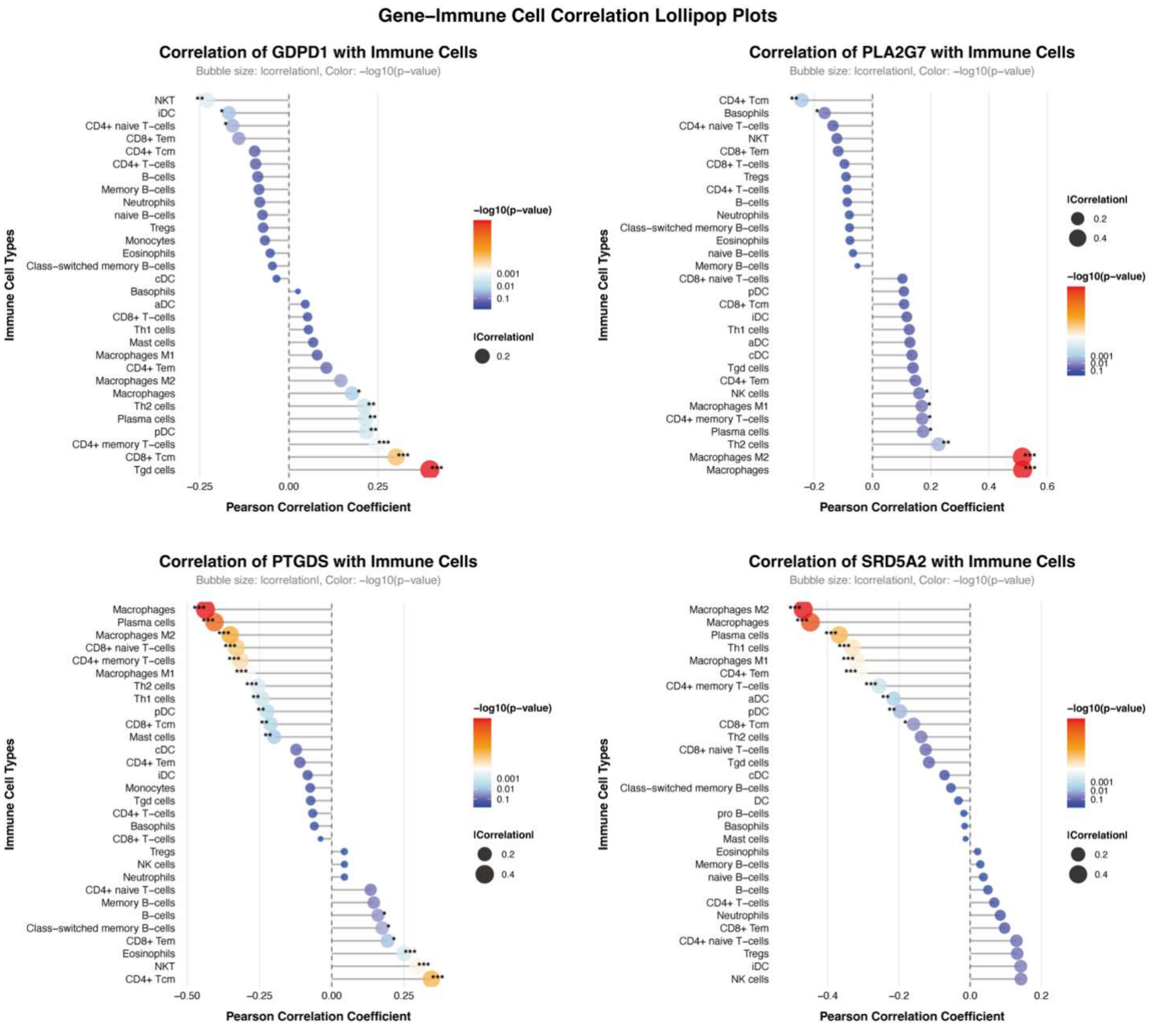

Figure S2. Correlation analysis of four metabolism-related hub genes with immune cells using the xCell method. (*$p < 0.05$, **$p < 0.01$, ***$p < 0.001$).

Table S1 Clinical characteristics of PCa patients from the GEO database.

Clinical characteristics	GSE220095	GSE70769
**Number of patients (%)**	176	94
BCR	75 (42.6)	40 (42.6)
Non-BCR	101 (57.4)	54 (57.4)
**Gleason (%)**		
< 7	35 (19.9)	20 (21.3)
= 7	120 (68.2)	56 (59.6)
> 7	21 (11.9)	15 (15.9)
Unknown	0 (0)	3 (3.2)
**T-Stage (%)**		
T1+T2	117 (66.5)	80 (85.1)
T3+T4	59 (33.5)	9 (9.6)
Unknown	0 (0)	5 (5.3)
**N-Stage (%)**		
N0	146 (83.0)	18 (19.1)
N1	22 (12.5)	0 (0)
Nx	8 (4.5)	76 (80.9)
**PSA (ng/ml, pre-biopsy) (%)**		
< 10	106 (60.2)	56 (59.6)
10-20	40 (22.7)	25 (26.6)
> 20	30 (17.1)	9 (9.6)
Unknown	0 (0)	4 (4.2)
